# Optimizing Dendritic Cell-Based Immunotherapy: Tackling the Complexity of Different Arms of the Immune System

**DOI:** 10.1155/2012/690643

**Published:** 2012-07-18

**Authors:** Ilse Van Brussel, Zwi N. Berneman, Nathalie Cools

**Affiliations:** ^1^Laboratory of Pharmacology, Department of Translational Pathophysiological Research, Faculty of Medicine and Health Sciences, University of Antwerp, Wilrijk, 2610 Antwerp, Belgium; ^2^Laboratory of Experimental Hematology, Vaccine and Infectious Disease Institute (VAXINFECTIO), Faculty of Medicine and Health Sciences, University of Antwerp, Wilrijk, 2610 Antwerp, Belgium

## Abstract

Earlier investigations have revealed a surprising complexity and variety in the range of interaction between cells of the innate and adaptive immune system. Our understanding of the specialized roles of dendritic cell (DC) subsets in innate and adaptive immune responses has been significantly advanced over the years. Because of their immunoregulatory capacities and because very small numbers of activated DC are highly efficient at generating immune responses against antigens, DCs have been vigorously used in clinical trials in order to elicit or amplify immune responses against cancer and chronic infectious diseases. A better insight in DC immunobiology and function has stimulated many new ideas regarding the potential ways forward to improve DC therapy in a more fundamental way. Here, we discuss the continuous search for optimal in vitro conditions in order to generate clinical-grade DC with a potent immunogenic potential. For this, we explore the molecular and cellular mechanisms underlying adequate immune responses and focus on most favourable DC culture regimens and activation stimuli in humans. We envisage that by combining each of the features outlined in the current paper into a unified strategy, DC-based vaccines may advance to a higher level of effectiveness.

## 1. Introduction

Dendritic cells (DCs), originally described by Steinman and Cohn [1], serve as a crucial link between innate and adaptive immunity [2]. Although they represent only a small population of leukocytes, they are the most powerful antigen presenting cells (APC) with the unique ability to activate naive T cells [3]. As sentinel members of the *innate immune system*, DCs respond to antigens and molecules containing pathogen-associated molecular patterns (PAMPs) or damage-associated molecular patterns (DAMPs)—so-called danger signals—by the generation of protective cytokines [4]. As members of the *acquired immune system*, DCs respond to these harmful molecules by efficient antigen uptake, processing, and presentation, and hence DCs are crucial in the initiation of adaptive immune responses. Besides their potent capacity to stimulate naive T cells, effector T cells and memory T cells, as well as B cells, they are also involved in the maintenance of tolerance against harmless (auto)antigens [4, 5].

Because of their immunoregulatory capacities and because very small numbers of activated DCs are highly efficient at generating immune responses against antigens [6], DCs have been vigorously used in clinical trials in order to elicit or amplify immune responses against cancer and chronic infectious diseases [7]. Although an impressive amount of data has been obtained from these clinical trials completed thus far, the outcomes were not in line with initial expectations [8–10]. A critical issue in the development of DC-based vaccines is that their ability to stimulate immune responses depends largely on the activation state of DCs. In this paper, we discuss the continuous quest for the best in vitro conditions in order to generate clinical-grade DCs with a potent immunogenic potential. For this, we explore the molecular and cellular mechanisms underlying adequate immune responses and focus on optimal DC culture regimens and activation stimuli in humans.

## 2. Origin and Subsets of DCs

DCs originate from CD34^+^ haematopoietic stem cells in the bone marrow and circulate as precursors through the blood stream to target tissues. Additionally, it is well established that during physiological stress, monocytes are also a source of DC precursors and differentiate into immature DCs in the presence of GM-CSF and a variety of other cytokines. Immature DCs take residence at sites of potential antigen entry and are specialized in antigen capturing and processing. They recognize the so-called pathogen-associated molecular patterns (PAMPs) which are evolutionary conserved structures, including microbial lipids, carbohydrates, nucleic acids and intermediates of viral replication (double-stranded (ds)RNA), via pattern recognition receptors (PRRs) [11]. There are several types of PRRs that are involved in innate recognition of pathogens, including toll-like receptors (TLRs), nucleotide-binding-oligomerization-domain-(NOD-like) receptors, interferon (IFN-induced) dsRNA-activated protein kinase (PKR), and RIG-I-like helicases [12]. Once DCs have captured a foreign nonself-antigen, they undergo a highly regulated maturation process and remodel into fully activated antigen-presenting DCs [13] capable to elicit effective immune responses. Indeed, mature DCs express high levels of several costimulatory molecules as well as major histocompatibility complex (MHC) molecules on their surface [14]. Maturation of DCs also induces the production of chemokines that attract naive and memory T cells. During the maturation process, DCs exit the nonlymphoid tissues to migrate via afferent lymph to lymphoid tissues. Subsequently, mature DCs will activate (naive) T- and B-lymphocytes that recognize the presented antigen as peptide-MHC complexes on the surface of the DC. Yet, additionally, positive amplification of antigen presentation via costimulation and secretion of various cytokines is also crucial to induce proper immune responses [3, 15] (cfr. 3.1).

**Figure 1:**
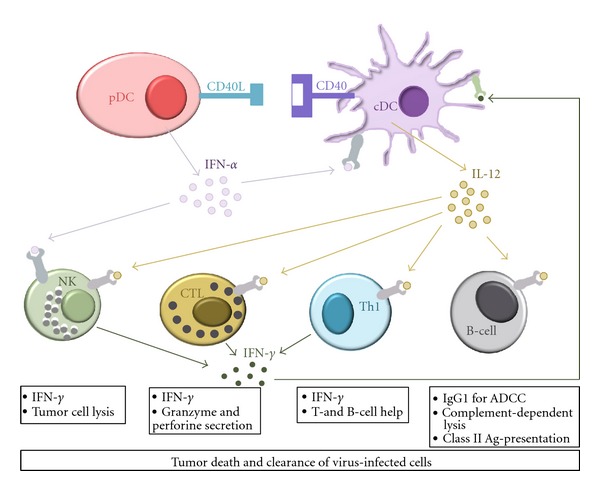
Cooperative action of different DC subsets to tackle both innate and adaptive immunity for clearance of tumors and viral infection.

Besides the above-delineated classical view of the DC life cycle, it has gradually become clear that DCs do not represent a homogeneous population. Briefly, the first division is the distinction between plasmacytoid and myeloid or conventional DCs (cDCs). Plasmacytoid DCs (pDCs), also referred to as type I IFN-producing cells (IPCs), are the key effectors in the innate immune system because of their extra-ordinary capacity to produce type I IFN upon viral infection [16, 17]. The conventional DCs can be further subdivided according to their localization: (i) lymphoid organ-resident DCs, (ii) peripheral tissue-resident DCs (e.g., langerhans cells and interstitial DCs), and (iii) circulating DCs. In human blood, differences in DC subsets can be identified based on differential expression of specific markers: pDCs express CD303 (BDCA-2), CD304 (BDCA-4), and CD123 (IL-3R*α*), whereas cDCs are characterized by their expression of CD1c (BDCA-1) and CD11c [18, 19]. In addition, pDCs and cDCs also express a different set of toll-like receptors TLRs [20]. In brief, pDCs express mainly TLR7 and TLR9, whereas cDCs exhibit strong expression of TLR1, TLR2, TLR3, TLR4, and TLR8. Accordingly, pDCs mainly recognize viral components and produce a large amount of IFN-*α*. In contrast, cDCs recognize bacterial components and produce proinflammatory cytokines such as TNF-*α*, IL-6, and IL-12p70 to activate proinflammatory T-cell subsets [T helper type 1 (Th1)/Th17] and consequently recruit cytotoxic T-lymphocytes (CTL). Because of the unique biological function of each DC subset, it was proposed that a specific DC lineage determines the outcome of T-cell contact, that is, tolerance or immunity. Indeed, it was initially thought that cDCs were inducers of immunity, while pDCs induced tolerance [21]. However, nowadays pDCs are believed to be the key effector cells in the early antiviral innate immune response by producing large amounts of type I interferons upon viral infection. Furthermore, it has been shown that pDCs augment immune responses by cross-talking with cDCs by the production of IFN-*α*, thereby playing a key role in effective stimulation of adaptive immunity as well. In addition to IFN-*α*  production, it has been demonstrated that mouse pDCs also express CD40L, which activates cDCs to produce IL-12p70 [22] (Figure 1).

Recently, several groups identified a unique human DC subset (CD11c^+^BDCA-3^+^) as the homologue of mouse CD8*α* ^+^ DC [23–26]. Of particular importance is their superior antigen cross-presentation capacity, expression of the XC chemokine receptor 1 (XCR1), and their capacity to produce high levels of bioactive IL-12p70. Initially, it was suggested that BDCA-3^+^ DCs and BDCA-1^+^ DCs may represent maturational stages of the same cell type. The fact that BDCA-3 expression is induced on a reasonable proportion of BDCA-1^+^ DCs after culture-induced maturation may be considered an argument in favour of the former concept. However, since the same observation was also made for IL-3-stimulated pDC, such data could also be taken as an argument in favour of a similar relationship between BDCA-3^+^ DCs and pDCs [27]. Nowadays, it is well accepted that BDCA-3^+^ DCs represent a unique myeloid DC subset that effectively activates CD8^+^ CTL, in analogy with mouse CD8*α* ^+^ DCs. This supports a potential key role for the myeloid BDCA-3^+^ DC subset in immunity to viruses, as well as other intracellular pathogens [28–30] and may have important implications in the design of human DC vaccines.

## 3. The Immune System against Cancer and ****Chronic Infectious Diseases

### 3.1. 3-Signal Theory for T-Cell Activation

Therapeutic vaccines to treat chronic infectious diseases (such as human immunodeficiency virus (HIV), cytomegalovirus (CMV), hepatitis B virus (HBV), and hepatitis C virus (HCV)), or numerous tumor types (including melanoma, leukaemia, breast, and prostate cancer) mainly aim to induce antigen-specific cell-mediated immunity to clear infected cells and eliminate tumor cells. Recent studies have shown that DCs play a critical role in directing effector T-cell responses towards a Th1, Th2, Th17, or regulatory T cell (Treg) response [31, 32]. Briefly, upon maturation, DCs upregulate the expression of certain products necessary to supply T lymphocytes with the 3 signals that will determine their activation status and general fate [33]: antigen-specific signalling via the T cell receptor (TCR), mediated by the binding of MHC-peptide complexes to the TCR drives the initial interaction between DCs and T cells (i.e., signal 1). Costimulation by surface molecules on APC, such as DCs, can either amplify or regulate the interaction with T cells (i.e., signal 2) [34]. Costimulatory molecules can be divided in two classes: Ig superfamily members, including CD28, that interact with several members of the B7 family (CD80/CD86) [35–37] on the one hand, and TNF receptor superfamily members, including CD27 and CD40, that bind to membrane-bound proteins of the TNF superfamily [35, 38, 39] on the other hand. CD28 is expressed on T cells, and is the receptor for CD80 and CD86 expressed on activated APC [37]. Ligation of CD28 provides costimulatory signals required for T cell activation: (i) altering the threshold level of TCR ligation required for activation, (ii) reducing the time needed to stimulate naive T cells and (iii) enhancing the magnitude of the T cell response. Without CD28 signalling, the T cell would either become apoptotic or anergic [40]. The B7 family of costimulatory molecules has been extensively reviewed elsewhere [41, 42]. CD27, a member of the TNF receptor superfamily, is constitutively expressed on the surface of naive T cells, in contrast to other members of the TNF receptor family [43] and can thus play a role during the initiation of T cell responses [35]. The contribution of CD27 to the immune response is dependent upon CD70 expression [44]. While during primary T cell activation there seems to be a certain redundancy in CD80/CD86 and CD70 costimulation, it is triggering of CD27 on T-lymphocytes by its ligand CD70 that enhances the magnitude of antigen-specific cytotoxic T cell reponses [38, 45], which is required for effective immunotherapy. CD27/CD70 interaction increases the initial expansion and survival of antigen-specific T cells [46] and improves their cytotoxic capacity [47]. Furthermore, a recent study has shown that CD70 expressed on mouse DEC205^+^ cDCs represents an IL-12p70-independent Th1-inducing factor [48] (vide infra). Taken together, enhancing CD70 expression on DCs would lead to the development of a vaccine strategy capable of facilitating the CD27/CD70 interaction, and hence the induction of an adequate anti-tumor or antiviral immune response. Finally, mature DCs can secrete a variety of pro- and anti-inflammatory cytokines for differentiation from naive T cells to effector T cells (i.e., signal 3). One well-studied third signal agent is interleukin (IL)-12p70 for the induction of Th1 and CTL [49], which are essential for efficient tumor/pathogen rejection [50]. IL-12p70 is a multifunctional proinflammatory cytokine with pleiotropic effects and comprises two subunits: p35 and p40. Highly-coordinated p35 and p40 gene expression results in the formation of the biologically active form IL-12p70 and is essential for initiation of an effective immune response. Indeed, IL-12p70 activates natural killer (NK) and T cells to produce mainly IFN-*γ*, it favours the generation of CTL and it enhances the cytotoxic activation of activated NK cells [51]. Besides the activation of innate and antigen-specific adaptive immunity against the tumor cells, the antitumor effects of IL-12p70 are based on the ability to inhibit tumor angiogenesis through IFN-*γ*  [52, 53]. In addition, IL-12p70 is crucial in the early phase of host defence against microbial infections [52, 54, 55], where it is produced within a few hours after bacterial, fungal or parasitic infection [52]. Thus, to develop an efficient vaccine against tumors or chronic infectious diseases, DCs producing the biologically active form IL-12p70 are desired [56].

### 3.2. Other Arms of Cellular Immunity Required to Fight Cancer and Chronic Infectious Diseases

Rather than simply recruiting Th1 cells and CTL, vaccines should be designed to recruit other cellular arms of the immune system as well, for example, NK cells and antibody-producing B cells. In this perspective, it has been shown that DCs also play a key role in the activation of NK cells that can have powerful effects against tumor cells, particularly those with attenuated MHC expression [57]. Indeed, in response to DC-derived cytokines, such as IL-12p70 and IL-18, NK cells are able to produce IFN-*γ*  [58]. In turn, exposure to signals provided by activated NK cells subsequently induces DCs to mature into a highly stimulatory phenotype that produces sustained IL-12p70, thereby promoting adaptive immunity [59, 60]. Overall, these findings support the concept to include DC-NK interactions in order to improve DC-based immunotherapy. Furthermore, recent studies that have resulted in reappraisal of the potential of antibodies in the control of tumors and viruses support the strategy that DC-based vaccines should also be designed with antibody production in mind [61–63]. In addition to priming of T cells and NK cells, the group of Banchereau have recently demonstrated that DCs may also directly signal naive B-cell differentiation through the production IL-12p70 [64] and indirectly by promoting the differentiation of IL-21-producing T follicular helper cells (Tfh) in an IL-12p70-dependent manner [65, 66]. These observations suggest that IL-12p70 could constitute a potent vaccine adjuvant in situations when both the cellular and humoral arms of the immune system are required, such as cancer [62, 63] and HIV [61]. Indeed, studies with rhesus macaques have concluded that IL-12p70 enhances the induction of specific antibody responses in vivo when used as vaccine adjuvant [67–69]. Noteworthy, IL-12p70 also possesses a number of powerful nonimmunologically related anticancer activities. For example, IL-12p70 plays a role as an antiangiogenic agent that can strongly inhibit the formation of neovasculature [53].

Taken together, the goal of many DC-based vaccination protocols is to cultivate DCs that are capable of expressing immunostimulatory cytokines (IL-12p70) and costimulatory molecules (CD70) in parallel with antigen presentation. Since expression of costimulatory molecules and cytokine secretion can be influenced by environmental signals during DC maturation, it is necessary to find an optimal cytokine environment for DC maturation in order to create a powerful vaccine against several cancer types or chronic infectious diseases. Various attempts have subsequently been made in order to harness DC to achieve most powerful immunity, including strategies to enhance or stabilise antigen-specific stimulation, as well as essential costimulatory modulation of DCs.

## 4. Harnessing DCs for Clinical Use

### 4.1. Antigen Loading Strategy

To maximize the efficiency and stability of antigen presentation by DCs, several strategies have been developed. These include direct in vivo delivery of antigen to circulating DCs in patients [70], as well as a variety of ways for in vitro loading of DCs with antigen. Indeed, antigens coupled to antibodies specific for DC markers, such as 33D1 or DEC-205, have already been used in preclinical models to deliver antigens to DCs in vivo [71]. Additionally, DCs transduced ex vivo with tumor- or viral-derived mRNA or DNA [72–74], fused with tumor cells [75, 76], or directly loaded with tumor- or viral-derived peptides [77, 78] have been tested for the induction of antigen-specific immune responses in vitro and in vivo. While the use of peptides as a source of antigen has several limitations when implementing clinical trials with antigen-loaded DCs, including human leukocyte antigen (HLA) restriction as well as a limited number of identified immunodominant tumor- and virus-associated antigens, we [79] and others [80–84] have previously shown that DCs transfected with mRNA-encoding antigens are superior to other loading strategies to induce immune responses. In general, there are several advantages regarding the use of mRNA for antigen loading of DCs [72, 85] as compared to tumor-associated peptides. mRNA transfection will generate multiple antigenic epitopes, possibly more immunogenic than those already characterized, independent of the patient's HLA haplotype. In addition, mRNA can be isolated and amplified from autologous tumor or virally infected cells in order to obtain mRNA encoding patient-specific antigens [86–88]. Moreover, because mRNA only has a short half-life and does not integrate in the host genome, genetic modification of DCs by mRNA electroporation is considered to be highly safe and an easily applicable clinical tool.

### 4.2. Different Sources for Isolation or Generation of DCs

The earliest studies on DC vaccination were initiated in 1993 and utilized whole blood leukapheresis products with subsequent gradient centrifugation procedures to enrich for rare immature DC precursors of the peripheral blood before antigen loading and maturation [89]. However, because of low yield of circulating DCs and difficulty to obtain them, the clinical utility of DC vaccines was initially limited. In a second attempt to directly isolate DCs from peripheral blood, they were first mobilized by cytokines such as Flt3-ligand [90, 91]. Unfortunately, the in vivo expanded cells lacked efficient protein uptake properties [89]. Moreover, although blood DCs from patients with a malignant or chronic infectious disease may seem to have normal distributions, they might have some functional defects, such as a lower expression of costimulatory molecules or an impaired capacity to stimulate autologous antigen-specific T cells [92, 93]. Currently, DCs for vaccination studies are generally obtained in large numbers after in vitro generation. At first, human DCs were cultured from CD34^+^ haematopoietic progenitors in the presence of granulocyte-macrophage colony stimulating factor (GM-CSF) and tumor necrosis factor alpha (TNF-*α*) [94, 95]. However, only few studies that used CD34^+^-derived DC preparations for vaccination protocols in clinical phase I studies have been reported [96, 97]. Nowadays, generating DCs from peripheral blood CD14^+^ monocytes is a generally-accepted method and is extensively used in experimental and clinical vaccination studies. In doing so, large numbers of monocyte-derived (mo-)DCs are obtained without necessity for pretreatment of donors with any cytokines to mobilize DC progenitor cells [98]. Yet, the design of DC-based clinical trials varies greatly, including DC preparation, and therefore, standardization and further improvement for clinical use are needed [99].

While a combination of granulocyte-macrophage colony-stimulating factor (GM-CSF) with IL-4 is most commonly used to induce immature DCs from monocytes [100, 101], a variety of other cytokines, such as IFN-*α*  [102–104], TNF-*α*  [105, 106], and IL-15 [107] have been used in combination with GM-CSF for this purpose. In this perspective, Santini et al. [102] as well as Arimoto-Miyamoto et al. [34] reported independently that IFN-*α*  induces rapid differentiation of freshly isolated GM-CSF-treated human monocytes into mo-DCs endowed with potent functional activities, both in vitro and in vivo [102, 103], possibly mediated by IFN-*α*-dependent induction of CD70 expression [34]. It must be noted however that IFN-*α*  also induces activity of RNases [108], and can not therefore be used in in vitro culture regimens for DCs when mRNA-based in vitro modification of DCs is wanted [109]. In addition, others have demonstrated that CD14^+^ monocytes respond to IL-15 by undergoing morphological transformation and acquiring characteristic DC features that facilitate antigen-specific responses of T cells [110]. In contrast to IFN-*α*-modulated DCs, mRNA electroporation appeared to serve as an efficient antigen-loading strategy for IL-15-treated DCs [111]. Furthermore, Chomarat et al. described that TNF-*α*  facilitates the induction of adaptive immunity also by promoting DC differentiation from CD14^+^ blood precursors in vitro [106]. However, it has been reported in contrast that TNF-*α*-treated semi mature DCs induce tolerance in experimental acute encephalitis (EAE), a mouse model for multiple sclerosis [112]. Moreover, due to strong plastic adherence before and to a lesser extent also after maturation, IL-15 and TNF-*α*  treatment for DC generation results in a lower DC yield [34]. Consequently, the well-established and generally used combination of GM-CSF and IL-4 [100, 101] is the most efficient method to obtain mo-DCs that express acceptable levels of CD70 with minimal loss of cells by adherence [34] and with good compatibility with a mRNA approach [79].

### 4.3. Various Stimuli to Obtain Mature DC

Regardless of how they are generated, it is important that DCs are activated to a mature phenotype, since immature DCs are no longer considered as competent candidates for vaccination trials because of their low T-cell activation potential [113–115]. Most DC culture regimens that have been commonly employed in clinical trials have activated DCs through the use of individual cytokines associated with inflammation [101] or inflammatory cytokine cocktails [116].

Indeed, in an attempt to resemble a physiological environment for DC maturation, balanced cocktails of maturation agents that may be the most representative of various inflammatory states have often been used. In 1996, Romani was the first to describe a method to mature DCs from human blood by using a conditioned medium containing an unidentified cytokine mixture produced by adherent peripheral blood mononuclear cells (PBMC) stimulated by human immunoglobulins or fixed *Staphylococcus aureus* Cowan I strain [98]. Only one year later, Morse and colleagues described a way to mature mo-DCs by adding TNF-*α*  to the culture medium. TNF-*α*  appeared to enhance the number of cells expressing the maturation marker CD83, which seemed to be the most potent allostimulatory cells in mixed lymphocyte reactions [117]. Also, Jonuleit et al. reported for the first time a well-defined cytokine cocktail to induce DC maturation, consisting of IL-1*β*, IL-6, TNF-*α*, and PGE_2_ [116]. This combination of proinflammatory mediators represents current “golden” standard for activation of DCs, although the concentration of the diverse components varies among studies. Fully-mature DCs induced by this combination of inflammatory cytokines have been consistently observed as superior to immature DCs in promoting a higher degree of specific T-cell priming in vitro and in vivo. While PGE_2_ increases the expression of CCR7 and hence the capacity of DCs to migrate to the regional lymph nodes through chemotaxis by CCL-19 and/or -21 [118], PGE_2_ also inhibits IL-12p70 secretion by DCs [56]. Although some details remain incompletely clarified, expression of IL-12p70 appears to be under unusually tight regulation and requires at least 2 signals activating both MyD88 (myeloid differentiation factor 88)- and TRIF (TIR domain-containing adapter-inducing IFN-*β*)-dependent pathways simultaneously for maximal expression [119, 120]. Of note, TLRs, commonly used for activation of DCs, are divided in those that are MyD88-dependent and those that are TRIF-dependent, hence explaining observed requirements of multiple TLR engagement for maximized IL-12p70 production. In this perspective, mature DCs with the potential to produce high amounts of biologically active IL-12p70 (10–15 ng/mL) were obtained by Mailliard et al. in 2004, who used a combination of IL-1*β*, TNF-*α*, IFN-*α*, IFN-*γ*, and poly I:C [121]. Although these mature mo-DCs displayed a slightly decreased migratory capacity [121], they induced significantly more antigen-specific cytotoxic T cells than did the “golden standard” counterparts, dependent on the high IL-12p70 secretion. In 2007, Zobywalski et al. proposed a cytokine cocktail consisting of TNF-*α*, IL-1*β*, IFN-*γ*, R848 and PGE_2_ as the best cocktail to allow large-scale processing of clinical-grade mo-DCs with the capacity to secrete IL-12p70 [56]. Addition of poly I:C to this cocktail significantly increased IL-12p70 production even more, yet it disabled the mature DCs to express the transgene after exogenous RNA electroporation and it led to a decline in cell viability [56]. Dohnal et al. used a mixture of LPS and IFN-*γ*  to mature DCs [122]. Although high IL-12p70 secretion by mature mo-DCs was previously attributed to the addition of IFN-*γ*  [121], IFN-*γ*  also appeared to be responsible for the low migratory ability of DCs cultivated in the presence of LPS and IFN-*γ*  [122, 123]. Nevertheless, this migratory problem could be fixed by including PGE_2_ in the maturation-inducing cytokine cocktail [123, 124]. In addition, whereas DC maturation by TLR ligand alone (including LPS, CpG, and poly I:C) has been reported to increase expression of classical activation markers as well as many inflammatory cytokines [125], a TLR agonist alone does not result in a substantial CD8^+^ T-cell response, which is probably due to no or very low levels of IL-12p70 secretion as well as insufficient induction of CD70 by TLR ligand stimulation alone [126]. According to Sanchez et al., expression of CD70 on mo-DCs requires combined TLR/CD40 stimulation [125]. In preliminary experiments, we experienced that addition of IFN-*γ*  alone to a cocktail of proinflammatory cytokines is neither enough for optimal CD70 induction on mo-DC (unpublished data). In contrast, addition of IFN-*γ*  in combination with the TLR7/8 agonist R848 to the standard maturation cocktail from which IL-6 was omitted resulted in a significant increase in CD70 expression (unpublished data).

From the above-mentioned observations, it may be evident that each compound added to a cytokine cocktail can influence DC phenotype and function in its own way and the “ideal” maturation mixture still needs to be well considered. Taken together, the “ideal” maturation cocktail to prime Th1-polarizing mo-DCs must contain PGE_2_ [123, 124], for its migration-inducing potential, a TLR ligand (e.g., LPS [125] or R848 (own unpublished data), but not poly I:C [56]) in combination with CD40L [125] or IFN-*γ*  [56], and some proinflammatory cytokines that have a positive impact on DC maturation (e.g., TNF-*α*  [117] and IL-1*β* [116]). In addition, the cocktail must be free of IL-6 which has been described to inhibit IL-12p70 secretion [34, 56], while IL-4 [126] and IL-10 [126] need to be eliminated from the cocktail as well, since these cytokines prevent CD70 expression.

Alternatively, one can optimise DC immunogenicity through molecular modification of the cells [109], for example, by selective overexpression of genes encoding immune-stimulatory signals (e.g., IL-12p70 [127, 128], CD40 or CD40 ligands [129, 130], and CD80/CD86) or by selective downmodulation of negative regulatory molecules, such as IL-10 [131, 132], IDO [133], SOCS1 [134, 135], and TGF-beta [136].

### 4.4. Influence of Different Oxygen Levels and Culture Media on Mo-DC Physiology

Mo-DC generation as well as maturation does not solely depend on the cytokine environment, but can also be influenced by oxygen levels, culture media and medium supplements. Mo-DCs are generally differentiated ex vivo in incubators that maintain atmospheric oxygen levels of 21% O_2_ in combination with 5% CO_2_. In contrast, DCs do not come across such high oxygen levels in vivo. Indeed, the oxygen levels in tissues are usually 3–5% [137], whereas approximately 12% in arterial blood [138]. In many inflamed and tumor tissues, even extremely low oxygen levels (<1%) have been found [139]. Therefore it is evident that DCs experience rapid changes of oxygen supply during their migration in different tissues. Although it is well recognized that tissue microenvironments are involved in regulating the development and function of immune cells, including B- and T cells, only few studies have investigated the effect of hypoxia (<1% oxygen saturation) or physiological oxygen levels (±3% oxygen saturation) on the differentiation of human DCs from progenitors and their maturation. Yang et al. reported that monocytes remain able to differentiate into DCs under hypoxia. However, these hypoxia-conditioned DCs displayed poor T cell-stimulatory activity and shifted towards a Th2-stimulatory phenotype [140], presumably as a consequence of the marked reduction of MHC class II and costimulatory molecule expression, [141] as well as of reduced Th1-polarizing cytokine secretion [140, 141]. The observed inhibition of DC function by hypoxia could possibly explain why most tumors can efficiently escape from host immune surveillance. However, Wang et al. showed only one year later that reoxygenation of hypoxia-differentiated DCs results in complete recovery of their mature phenotype and function, including a strong ability of the reoxygenated DCs to drive immune responses towards a proinflammatory Th1/Th17 direction [141]. Besides hypoxic conditions, one study investigated the influence of physiological oxygen levels on DC physiology and antigen-presenting capacity. Surprisingly, no difference in expression of surface molecules (CD54, CD40, CD83, CD86, HLA-DR, CXCR4, CCR7) nor secretion of TNF-*α*, IL-6, and IL-10 was observed between DC cultures under physiological (3%) or atmospherical (21%) oxygen levels [138]. Albeit that DCs stimulated with LPS or CD40L under physiological O_2_ conditions secreted higher amounts of IL-12p70, these DCs did not elicit increased CD8^+^ T-cell responses in vitro, as measured by IFN-*γ*  secretion [138]. Taken together, there is still some controversy on whether physiologically or atmospherically oxygen levels must be used for DC culture and not enough data exist to robustly support a conclusion.

For optimal production of clinical-grade DCs from peripheral blood monocytes, it is also important to choose the appropriate culture medium as well as potential serum supplements. Initially, most mo-DCs used for clinical trials were generated in medium supplemented with plasma or serum, such as fetal bovine serum (FBS) containing xenologous proteins. For this, FBS can be immunogenic and possibly transfer bovine-related infections, including bovine spongiform encephalopathy [98, 142]. However also the use of autologous or allogeneic (pooled) serum derived from patients or healthy controls, respectively, might lead to undesired immunomodulatory ingredients that can affect DC phenotype and function [143]. Therefore, it is clear that by eliminating the need for serum, an undesirable variable is removed making the medium more defined and consistent [142, 143]. For this reason, several clinical-grade serum-free media are now commercially available and have been tested, including XVivo15, XVivo20, and AIMV [98, 144, 145]. Although so far only a small amount of studies have compared mo-DCs differentiated in serum-free medium with cells cultured in medium containing serum, they all agree that serum-containing media were more able to generate mature mo-DCs as compared with serum-free media [143, 145, 146]. The latter resulted mainly in the generation of semimature mo-DCs that express CD83 (a mature DC marker [147]) as well as CD1a (an immature DC marker [98, 101]), and were slightly but consistently less able to produce IL12p70 in response to maturation-inducing stimuli [142, 143]. Other characteristics, including yield, surface expression of maturation markers, in vitro survival, migratory capacities and induction of lymphocyte proliferation, were comparable between DCs differentiated in serum-free or serum-containing media [99, 143, 145, 146]. In vivo assays following transfer of such mo-DCs generated in serum-free medium into humans are needed to decide whether the limited difference in CD1a expression and cytokine production is of true biological relevance.

## 5. Taking DC into the Clinic

### 5.1. Completed and Ongoing Clinical Trials

Despite the use of mature DCs in vaccination trials, results from multiple clinical trials with DC-based vaccines have been contradictory and only fractions of enrolled patients show potent antitumor or antiviral immune responses with moderate clinical response rates (approximately 10–15%) (reviewed in [10, 148–151]) or partial control of viremia and immune reconstitution [77, 152–154], respectively. Several studies suggested that this is because of inefficient activation of Th1-polarized responses due to incomplete DC maturation [155–157]. For this, different strategies are currently being pursued in order to improve the efficacy and outcome of DC-based cancer vaccines. Considering the above-mentioned powerful immune-stimulatory properties possessed by IL-12p70, DC-based vaccination strategies may consistently benefit from incorporation or endogenous induction of this cytokine. In a first phase I clinical trial by the group of Czerniecki [158], 13 breast cancer subjects were injected intranodally with short-term DCs activated with a cytokine-cocktail consisting of IFN-*γ*  and LPS in order to induce IL-12p70-secreting DCs. The authors reported induction of robust detectable immunity as evidenced by in vitro monitoring of circulating vaccine-induced antigen-specific CD4^+^ and CD8^+^ T cells, as well as both T-and B-cell infiltrates into tumor region as well as dramatic reductions in tumor volume. Additionally, Dohnal et al. [122] also showed the safety and feasibility of IFN-*γ*/LPS-activated DCs for the treatment of paediatric cancer patients. Besides that no adverse events were reported, they also demonstrated the potential of IL-12p70-secreting DCs to induce cellular immune responses. It should, however, be noted that Traxlmayr et al. [159] reported IL-12p70-dependent proliferation of immunosuppressive  *γδ*  T cells in cancer patients vaccinated with IL-12p70-secreting DCs, pointing to a negative regulatory feedback mechanism for DC-controlled immune responses.

Furthermore, it has been demonstrated by others [160–162] that DCs electroporated with mRNA encoding CD40 ligand, CD70, and constitutively active toll-like receptor 4, so-called TriMix DCs, display increased potential for the induction and amplification of tumor-specific responses in patients with advanced melanoma. Noteworthy, a positive delayed-type hypersensitivity assay (DTH) postvaccination correlated with Il-12p70 secreting capacity of vaccinated DCs.

### 5.2. Overcoming Tumor and Virus Immune Escape

One of the major obstacles against successful DC vaccination, is certain immunosuppressive mechanisms triggered by the tumor cells or viruses. Indeed, under the influence of the tumorigenic microenvironment, the host DCs may acquire a tolerogenic phenotype. These tumor-conditioned DCs could, in return, produce a variety of immunosuppressive molecules and thus further supporting tumor immune escape [163]. For example, many tumors produce IL-10 [164], a potent immunosuppressive cytokine. We (unpublished data) and others have previously shown that DC differentiation and functional activities are tightly regulated by this cytokine [165, 166]. In return, DCs can secrete IL-10 and effectively inhibit T-cell activation. Additionally, numerous viruses, including human CMV, HIV, herpes simplex virus type 1, and measles virus, target DCs [167, 168], and have evolved strategies to specifically modulate DC phenotype and/or function, thereby promoting virus-mediated immune escape. For example, DCs infected by human CMV are characterized by reduced expression of MHC class I and II molecules, costimulatory molecules, and proinflammatory cytokines, which consequently results in reduced T-cell activation [169]. Nowadays, emerging evidence indicates that one of the most effective ways to enhance the efficacy of DC-based immunotherapy is by targeting the negative arm of immune regulation. For future clinical trials, this may be achieved by the use of small interfering RNA (siRNA) for knocking down IL-10 expression by DCs [131, 132], or other negative regulatory molecules, such as indoleamine 2,3-dioxygenase (IDO) [133], suppressors of cytokine signalling 1 (SOCS1) [134, 135], and transforming growth factor (TGF)-beta [136]. Indeed, inhibition of expression of these regulatory molecules has been demonstrated to significantly enhance the abilities of DCs to present tumor antigens, to produce IL-12p70, and to induce effectively antitumor responses.

### 5.3. Future Perspectives

With respect to tackle different arms of the immune system, many different approaches are currently being pursued. In particular, considering the distinct ability of different DC subsets in inducing both innate and adaptive immunity, the exploitation of specific subsets of DCs to elicit the desired immune response is foreseen. Although pDCs primarily contribute to innate antiviral immune responses by producing IFN-*α*/*β*  [16], this ability also has been reported to activate other DCs, including those involved in cross-priming [170], and consequently greater activation of adaptive immune responses. In doing so, pDCs may play a critical role in provoking cancer immunity. Hence, combination therapies aiming at interaction of pDCs and cDCs to stimulate T-cell priming, and hence effective anti-tumor or antiviral immunity are needed in cancer patients and chronically infected patients (Figure 1).

Additionally, differentiation of monocytes into DCs with cocktails including GM-CSF and IL-15 will generate cells with the phenotype and characteristics of Langerhans cells (LC), which are far more efficient in vitro in priming antigen-specific CD8^+^ T cells than DCs derived with GM-CSF and IL-4 [111, 171]. As described by others, LCs are very efficient in cross-presenting peptides to CD8^+^ T cells, which acquire potent cytotoxicity and are able to efficiently kill target cells, including tumor cell lines that express peptide-HLA complex, only at low amounts [172] in an IL-15-dependent manner. The pivotal role of LC to allow maximal stimulation of both humoral and cellular immune responses, supports the important concept for targeting LC in the design of vaccines that aim at eliciting strong cellular immune responses [66, 173, 174].

The recent identification of human CD141^+^ DCs that can effectively cross-present antigens has clear implications for the design of new therapies to treat cancers and infectious diseases with improved efficacy. It has been reported that the limiting cytokine for the development of the murine counterpart is the Fms-related tyrosine kinase 3 ligand (Flt3L), rather than GM-CSF or M-CSF, which has major influence on the development of inflammatory and migratory DCs [175–177]. However, although Poulin et al. [24] made a first attempt to delineate in vitro culturing conditions for the generation of CD141^+^ DCs from human progenitor cells, further optimization of such protocols is necessary to allow for their use in adoptive transfer immunotherapy approaches.

## 6. Conclusion

Current efforts for DC-based modalities have been compromised by a failure to utilize the full potential of DCs. However, even though only limited success rates have been achieved to date, DC vaccination remains a promising immunological approach against tumors and/or viruses and deserves further exploration. Alternative strategies to enhance DC immunogenicity by functional conditioning and molecular modifications have been investigated in vitro. The different findings discussed here, indicate that DCs can indeed be functionally conditioned and genetically modified to acquire an enhanced immunogenic phenotype. For this, time has come to bring DC-based immunotherapy to the next level and implement above-mentioned observations in a standardized regimen for alternatively conditioned DCs. Results from first clinical trials will subsequently reveal their potential in order to improve treatment of cancer and chronic infections.

## References

[1] Steinman RM, Cohn ZA (1973). Identification of a novel cell type in peripheral lymphoid organs of mice. I. Morphology, quantitation, tissue distribution. *The Journal of Experimental Medicine*.

[2] Banchereau J, Steinman RM (1998). Dendritic cells and the control of immunity. *Nature*.

[3] Banchereau J, Briere F, Caux C (2000). Immunobiology of dendritic cells. *Annual Review of Immunology*.

[4] Granucci F, Zanoni I, Ricciardi-Castagnoli P (2008). Central role of dendritic cells in the regulation and deregulation of immune responses. *Cellular and Molecular Life Sciences*.

[5] Steinman RM (2007). Dendritic cells: versatile controllers of the immune system. *Nature Medicine*.

[6] Figdor CG, de Vries IJM, Lesterhuis WJ, Melief CJM (2004). Dendritic cell immunotherapy: mapping the way. *Nature Medicine*.

[7] Steinman RM, Banchereau J (2007). Taking dendritic cells into medicine. *Nature*.

[8] Bodey B, Bodey B, Siegel SE, Kaiser HE (2000). Failure of cancer vaccines: the significant limitations of this approach to immunotherapy. *Anticancer Research*.

[9] Andrews DM, Maraskovsky E, Smyth MJ (2008). Cancer vaccines for established cancer: how to make them better?. *Immunological Reviews*.

[10] Schuler G (2010). Dendritic cells in cancer immunotherapy. *European Journal of Immunology*.

[11] Mogensen TH (2009). Pathogen recognition and inflammatory signaling in innate immune defenses. *Clinical Microbiology Reviews*.

[12] Kumagai Y, Takeuchi O, Akira S (2008). Pathogen recognition by innate receptors. *Journal of Infection and Chemotherapy*.

[13] van Vré EA, van Brussel I, Bosmans JM, Vrints CJ, Bult H (2011). Dendritic cells in human atherosclerosis: from circulation to atherosclerotic plaques. *Mediators of Inflammation*.

[14] Lechmann M, Zinser E, Golka A, Steinkasserer A (2002). Role of CD83 in the immunomodulation of dendritic cells. *International Archives of Allergy and Immunology*.

[15] Sallusto F, Lanzavecchia A (2002). The instructive role of dendritic cells on T-cell responses. *Arthritis Research*.

[16] Cella M, Jarrossay D, Faccheth F (1999). Plasmacytoid monocytes migrate to inflamed lymph nodes and produce large amounts of type I interferon. *Nature Medicine*.

[17] Siegal FP, Kadowaki N, Shodell M (1999). The nature of the principal type 1 interferon-producing cells in human blood. *Science*.

[18] MacDonald KPA, Munster DJ, Clark GJ, Dzionek A, Schmitz J, Hart DNJ (2002). Characterization of human blood dendritic cell subsets. *Blood*.

[19] Ju X, Clark G, Hart DN (2010). Review of human DC subtypes. *Methods in Molecular Biology*.

[20] Kadowaki N, Ho S, Antonenko S (2001). Subsets of human dendritic cell precursors express different toll-like receptors and respond to different microbial antigens. *Journal of Experimental Medicine*.

[21] Kronin V, Fitzmaurice CJ, Caminschi I, Shortman K, Jackson DC, Brown LE (2001). Differential effect of CD8^+^ and CD8^−^ dendritic cells in the stimulation of secondary CD4^+^ T cells. *International Immunology*.

[22] Krug A, Towarowski A, Britsch " S (2001). Toll-like receptor expression reveals CpG DNA as a unique microbial stimulus for plasmacytoid dendritic cells which synergizes with CD40 ligand to induce high amounts of IL-12. *European Journal of Immunology*.

[23] Jongbloed SL, Kassianos AJ, McDonald KJ (2010). Human CD141^+^ (BDCA-3)^+^ dendritic cells (DCs) represent a unique myeloid DC subset that cross-presents necrotic cell antigens. *Journal of Experimental Medicine*.

[24] Poulin LF, Salio M, Griessinger E (2010). Characterization of human DNGR-1^+^ BDCA3^+^ leukocytes as putative equivalents of mouse CD8*α*
^+^ dendritic cells. *Journal of Experimental Medicine*.

[25] Crozat K, Guiton R, Contreras V (2010). The XC chemokine receptor 1 is a conserved selective marker of mammalian cells homologous to mouse CD8*α*
^+^ dendritic cells. *Journal of Experimental Medicine*.

[26] Bachem A, Güttler S, Hartung E (2010). Superior antigen cross-presentation and XCR1 expression define human CD11c^+^CD141^+^ cells as homologues of mouse CD8^+^ dendritic cells. *Journal of Experimental Medicine*.

[27] Dzionek A, Fuchs A, Schmidt P (2000). BDCA-2, BDCA-3, and BDCA-4: three markers for distinct subsets of dendritic cells in human peripheral blood. *Journal of Immunology*.

[28] Allan RS, Smith CM, Belz GT (2003). Epidermal viral immunity induced by CD8*α*
^+^ dendritic cells but not by langerhans cells. *Science*.

[29] Belz GT, Smith CM, Eichner D (2004). Cutting edge: conventional CD8*α*
^+^ dendritic cells are generally involved in priming CTL immunity to viruses. *Journal of Immunology*.

[30] Lukens MV, Kruijsen D, Coenjaerts FEJ, Kimpen JLL, van Bleek GM (2009). Respiratory syncytial virus-induced activation and migration of respiratory dendritic cells and subsequent antigen presentation in the lung-draining lymph node. *Journal of Virology*.

[31] Cools N, Ponsaerts P, van Tendeloo VFI, Berneman ZN (2007). Balancing between immunity and tolerance: an interplay between dendritic cells, regulatory T cells, and effector T cells. *Journal of Leukocyte Biology*.

[32] Cools N, Petrizzo A, Smits E (2011). Dendritic cells in the pathogenesis and treatment of human diseases: a Janus Bifrons?. *Immunotherapy*.

[33] Kaliński P, Hilkens CMU, Wierenga EA, Kapsenberg ML (1999). T-cell priming by type-1 and type-2 polarized dendritic cells: the concept of a third signal. *Immunology Today*.

[34] Arimoto-Miyamoto K, Kadowaki N, Kitawaki T, Iwata S, Morimoto C, Uchiyama T (2010). Optimal stimulation for CD70 induction on human monocyte-derived dendritic cells and the importance of CD70 in naive CD4^+^ T-cell differentiation. *Immunology*.

[35] Rowley TF, Al-Shamkhani A (2004). Stimulation by soluble CD70 promotes strong primary and secondary CD8^+^ cytotoxic T cell responses in vivo. *Journal of Immunology*.

[36] Frauwirth KA, Thompson CB (2002). Activation and inhibition of lymphocytes by costimulation. *Journal of Clinical Investigation*.

[37] Sharpe AH, Freeman GJ (2002). The B7-CD28 superfamily. *Nature Reviews Immunology*.

[38] Croft M (2003). Costimulation of T cells by OX40, 4-1BB, and CD27. *Cytokine and Growth Factor Reviews*.

[39] Watts TH, DeBenedette MA (1999). T cell co-stimulatory molecules other than CD28. *Current Opinion in Immunology*.

[40] Alegre ML, Frauwirth KA, Thompson CB (2001). T-cell regulation by CD28 and CTLA-4. *Nature Reviews Immunology*.

[41] Collins M, Ling V, Carreno BM (2005). The B7 family of immune-regulatory ligands. *Genome Biology*.

[42] Carreno BM, Collins M (2002). The B7 family of ligands and its receptors: new pathways for costimulation and inhibition of immune responses. *Annual Review of Immunology*.

[43] Croft M (2003). Co-stimulatory members of the TNFR family: keys to effective T-cell immunity?. *Nature Reviews Immunology*.

[44] Tesselaar K, Xiao Y, Arens R (2003). Expression of the murine CD27 ligand CD70 in vitro and in vivo. *Journal of Immunology*.

[45] Taraban VY, Rowley TF, Al-Shamkhani A (2004). Cutting edge: a critical role for CD70 in CD8 T cell priming by CD40-licensed APCs. *Journal of Immunology*.

[46] Dolfi DV, Katsikis PD (2007). CD28 and CD27 costimulation of CD8^+^ T cells: a story of survival. *Advances in Experimental Medicine and Biology*.

[47] Yamada S, Shinozaki K, Agematsu K (2002). Involvement of CD27/CD70 interactions in antigen-specific cytotoxic T-lymphocyte (CTL) activity by perforin-mediated cytotoxicity. *Clinical and Experimental Immunology*.

[48] Soares H, Waechter H, Glaichenhaus N (2007). A subset of dendritic cells induces CD4^+^ T cells to produce IFN-*γ* by an IL-12-independent but CD70-dependent mechanism in vivo. *Journal of Experimental Medicine*.

[49] Trinchieri G, Pflanz S, Kastelein RA (2003). The IL-12 family of heterodimeric cytokines: new players in the regulation of T cell responses. *Immunity*.

[50] Cho HJ, Takabayashi K, Cheng PM (2000). Immunostimulatory DNA-based vaccines reduce cytotoxic lymphocyte activity by a T-helper cell-independent mechanism. *Nature Biotechnology*.

[51] Hensler T, Heidecke CD, Hecker H (1998). Increased susceptibility to postoperative sepsis in patients with impaired monocyte IL-12 production. *Journal of Immunology*.

[52] Trinchieri G, Scott P (1999). Interleukin-12: basic principles and clinical applications. *Current Topics in Microbiology and Immunology*.

[53] Voest EE, Kenyon BM, O’Reilly MS, Truitt G, D’Amato RJ, Folkman J (1995). Inhibition of angiogenesis in vivo by interleukin 12. *Journal of the National Cancer Institute*.

[54] Romani L, Puccetti P, Bistoni F (1997). Interleukin-12 in infectious diseases. *Clinical Microbiology Reviews*.

[55] van der Poll T, van Deventer SJH (1999). Cytokines and anticytokines in the pathogenesis of sepsis. *Infectious Disease Clinics of North America*.

[56] Zobywalski A, Javorovic M, Frankenberger B (2007). Generation of clinical grade dendritic cells with capacity to produce biologically active IL-12p70. *Journal of Translational Medicine*.

[57] Vivier E, Tomasello E, Baratin M, Walzer T, Ugolini S (2008). Functions of natural killer cells. *Nature Immunology*.

[58] Zitvogel L, Terme M, Borg C, Trinchieri G (2005). Dendritic cell-NK cell cross-talk: regulation and physiopathology. *Current Topics in Microbiology and Immunology*.

[59] Kalinski P, Giermasz A, Nakamura Y (2005). Helper role of NK cells during the induction of anticancer responses by dendritic cells. *Molecular Immunology*.

[60] Kalinski P, Mailliard RB, Giermasz A (2005). Natural killer-dendritic cell cross-talk in cancer immunotherapy. *Expert Opinion on Biological Therapy*.

[61] Chehimi J, Paganin C, Frank I, Chouaib S, Starr S, Trinchieri G (1995). Interleukin-12 in the pathogenesis and therapy of HIV disease. *Research in Immunology*.

[62] Vollmers HP, Brändlein S (2009). Natural antibodies and cancer. *New Biotechnology*.

[63] Mou Z, He Y, Wu Y (2009). Immunoproteomics to identify tumor-associated antigens eliciting humoral response. *Cancer Letters*.

[64] Dubois B, Massacrier C, Vanbervliet B (1998). Critical role of IL-12 in dendritic cell-induced differentiation of naive B lymphocytes. *Journal of Immunology*.

[65] Schmitt N, Morita R, Bourdery L (2009). Human dendritic cells induce the differentiation of interleukin-21-producing T follicular helper-like cells through interleukin-12. *Immunity*.

[66] Banchereau J, Klechevsky E, Schmitt N, Morita R, Palucka K, Ueno H (2009). Harnessing human dendritic cell subsets to design novel vaccines. *Annals of the New York Academy of Sciences*.

[67] Hirao LA, Wu L, Khan AS (2008). Combined effects of IL-12 and electroporation enhances the potency of DNA vaccination in macaques. *Vaccine*.

[68] Schadeck EB, Sidhu M, Egan MA (2006). A dose sparing effect by plasmid encoded IL-12 adjuvant on a SIVgag-plasmid DNA vaccine in rhesus macaques. *Vaccine*.

[69] van der Meide PH, Villinger F, Ansari AA (2002). Stimulation of both humoral and cellular immune responses to HIV-1 gp120 by interleukin-12 in Rhesus macaques. *Vaccine*.

[70] Tacken PJ, de Vries IJM, Torensma R, Figdor CG (2007). Dendritic-cell immunotherapy: from ex vivo loading to in vivo targeting. *Nature Reviews Immunology*.

[71] Unger WWJ, van Kooyk Y (2011). ’Dressed for success’ C-type lectin receptors for the delivery of glyco-vaccines to dendritic cells. *Current Opinion in Immunology*.

[72] Ponsaerts P, van Tendeloo VFI, Berneman ZN (2003). Cancer immunotherapy using RNA-loaded dendritic cells. *Clinical and Experimental Immunology*.

[73] Boudreau JE, Bonehill A, Thielemans K, Wan Y (2011). Engineering dendritic cells to enhance cancer immunotherapy. *Molecular Therapy*.

[74] Shurin MR, Gregory M, Morris JC, Malyguine AM (2010). Genetically modified dendritic cells in cancer immunotherapy: a better tomorrow?. *Expert Opinion on Biological Therapy*.

[75] Cathelin D, Nicolas A, Bouchot A (2011). Dendritic cell-tumor cell hybrids and immunotherapy: what's next?. *Cytotherapy*.

[76] Koido S, Hara E, Homma S, Ohkusa T, Gong J, Tajiri H (2009). Cancer immunotherapy by fusions of dendritic cells and tumor cells. *Immunotherapy*.

[77] Connolly NC, Whiteside TL, Wilson C, Kondragunta V, Rinaldo CR, Riddler SA (2008). Therapeutic immunization with human immunodeficiency virus type 1 (HIV-1) peptide-loaded dendritic cells is safe and induces immunogenicity in HIV-1-infected individuals. *Clinical and Vaccine Immunology*.

[78] Brody JD, Engleman EG (2004). DC-based cancer vaccines: lessons from clinical trials. *Cytotherapy*.

[79] van Tendeloo VFI, Ponsaerts P, Lardon F (2001). Highly efficient gene delivery by mRNA electroporation in human hematopoietic cells: superiority to lipofection and passive pulsing of mRNA and to electroporation of plasmid cDNA for tumor antigen loading of dendritic cells. *Blood*.

[80] Boczkowski D, Nair SK, Snyder D, Gilboa E (1996). Dendritic cells pulsed with RNA are potent antigen-presenting cells in vitro and in vivo. *Journal of Experimental Medicine*.

[81] Kavanagh DG, Kaufmann DE, Sunderji S (2006). Expansion of HIV-specific CD4^+^ and CD8^+^ T cells by dendritic cells transfected with mRNA encoding cytoplasm- or lysosome-targeted Nef. *Blood*.

[82] Melhem NM, Liu XD, Boczkowski D, Gilboa E, Barratt-Boyes SM (2007). Robust CD4^+^ and CD8^+^ T cell responses to SIV using mRNA-transfected DC expressing autologous viral Ag. *European Journal of Immunology*.

[83] Sæbøe-Larssen S, Fossberg E, Gaudernack G (2002). mRNA-based electrotransfection of human dendritic cells and induction of cytotoxic T lymphocyte responses against the telomerase catalytic subunit (hTERT). *Journal of Immunological Methods*.

[84] Strobel I, Berchtold S, Götze A, Schulze U, Schuler G, Steinkasserer A (2000). Human dendritic cells transfected with either RNA or DNA encoding influenza matrix protein M1 differ in their ability to stimulate cytotoxic T lymphocytes. *Gene Therapy*.

[85] van Driessche A, Ponsaerts P, van Bockstaele DR, van Tendeloo VFI, Berneman ZN (2005). Messenger RNA electroporation: an efficient tool in immunotherapy and stem cell research. *Folia Histochemica et Cytobiologica*.

[86] van Gulck ERA, Ponsaerts P, Heyndrickx L (2006). Efficient stimulation of HIV-1-specific T cells using dendritic cells electroporated with mRNA encoding autologous HIV-1 Gag and Env proteins. *Blood*.

[87] van Gulck ER, Vanham G, Heyndrickx L (2008). Efficient in vitro expansion of human immunodeficiency virus (HIV)-specific T-cell responses by gag mRNA-electroporated dendritic cells from treated and untreated HIV type 1-infected individuals. *Journal of Virology*.

[88] Boczkowski D, Nair SK, Nam JH, Lyerly HK, Gilboa E (2000). Induction of tumor immunity and cytotoxic T lymphocyte responses using dendritic cells transfected with messenger RNA amplified from tumor cells. *Cancer Research*.

[89] Reichardt VL, Brossart P, Kanz L (2004). Dendritic cells in vaccination therapies of human malignant disease. *Blood Reviews*.

[90] Morse MA, Nair S, Fernandez-Casal M (2000). Preoperative mobilization of circulating dendritic cells by Flt3 ligand administration to patients with metastatic colon cancer. *Journal of Clinical Oncology*.

[91] Maraskovsky E, Daro E, Roux E (2000). In vivo generation of human dendritic cell subsets by Flt3 ligand. *Blood*.

[92] Brown RD, Pope B, Murray A (2001). Dendritic cells from patients with myeloma are numerically normal but functionally defective as they fail to up-regulate CD80 (B7-1) expression after huCD40LT stimulation because of inhibition by transforming growth factor-*β*1 and interleukin-10. *Blood*.

[93] Ratta M, Fagnoni F, Curti A (2002). Dendritic cells are functionally defective in multiple myeloma: the role of interleukin-6. *Blood*.

[94] Caux C, Dezutter-Dambuyant C, Schmit D, Banchereau J (1992). GM-CSF and TNF-*α* cooperate in the generation of dendritic Langerhans cells. *Nature*.

[95] Lardon F, Snoeck HW, Berneman ZN (1997). Generation of dendritic cells from bone marrow progenitors using GM-CSF, TNF-*α*, and additional cytokines: antagonistic effects of IL-4 and IFN-*γ* and selective involvement of TNF-*α* receptor-1.. *Immunology*.

[96] Banchereau J, Palucka AK, Dhodapkar M (2001). Immune and clinical responses in patients with metastatic melanoma to CD34^+^ progenitor-derived dendritic cell vaccine. *Cancer Research*.

[97] Titzer S, Christensen O, Manzke O (2000). Vaccination of multiple myeloma patients with idiotype-pulsed dendritic cells: immunological and clinical aspects. *British Journal of Haematology*.

[98] Romani N, Reider D, Heuer M (1996). Generation of mature dendritic cells from human blood An improved method with special regard to clinical applicability. *Journal of Immunological Methods*.

[99] Tkachenko N, Wojas K, Tabarkiewicz J, Rolinski J (2005). Generation of dendritic cells from human peripheral blood monocytes—comparison of different culture media. *Folia Histochemica et Cytobiologica*.

[100] Sallusto F, Lanzavecchia A (1994). Efficient presentation of soluble antigen by cultured human dendritic cells is maintained by granulocyte/macrophage colony-stimulating factor plus interleukin 4 and downregulated by tumor necrosis factor *α*. *Journal of Experimental Medicine*.

[101] Zhou LJ, Tedder TF (1996). CD14^+^ blood monocytes can differentiate into functionally mature CD83^+^ dendritic cells. *Proceedings of the National Academy of Sciences of the United States of America*.

[102] Santini SM, Lapenta C, Logozzi M (2000). Type I interferon as a powerful adjuvant for monocyte-derived dendritic cell development and activity in vitro and in Hu-PBL-SCID mice. *Journal of Experimental Medicine*.

[103] Della Bella S, Nicola S, Riva A, Biasin M, Clerici M, Villa ML (2004). Functional repertoire of dendritic cells generated in granulocyte macrophage-colony stimulating factor and interferon-*α*. *Journal of Leukocyte Biology*.

[104] Korthals M, Safaian N, Kronenwett R (2007). Monocyte derived dendritic cells generated by IFN-*α* acquire mature dendritic and natural killer cell properties as shown by gene expression analysis. *Journal of Translational Medicine*.

[105] Iwamoto S, Iwai SI, Tsujiyama K (2007). TNF-*α* drives human CD14^+^ monocytes to differentiate into CD70^+^ dendritic cells evoking Th1 and Th17 responses. *Journal of Immunology*.

[106] Chomarat P, Dantin C, Bennett L, Banchereau J, Palucka AK (2003). TNF skews monocyte differentiation from macrophages to dendritic cells. *Journal of Immunology*.

[107] Mohamadzadeh M, Berard F, Essert G (2001). Interleukin 15 skews monocyte differentiation into dendritic cells with features of langerhans cells. *Journal of Experimental Medicine*.

[108] Samuel CE (2001). Antiviral actions of interferons. *Clinical Microbiology Reviews*.

[109] Boczkowski D, Nair S (2010). RNA as performance-enhancers for dendritic cells. *Expert Opinion on Biological Therapy*.

[110] Saikh KU, Khan AS, Kissner T, Ulrich RG (2001). IL-15-induced conversion of monocytes to mature dendritic cells. *Clinical and Experimental Immunology*.

[111] Anguille S, Smits ELJM, Cools N, Goossens H, Berneman ZN, van Tendeloo VFI (2009). Short-term cultured, interleukin-15 differentiated dendritic cells have potent immunostimulatory properties. *Journal of Translational Medicine*.

[112] Menges M, Rößner S, Voigtländer C (2002). Repetitive injections of dendritic cells matured with tumor necrosis factor *α* induce antigen-specific protection of mice from autoimmunity. *Journal of Experimental Medicine*.

[113] McIlroy D, Gregoire M (2003). Optimizing dendritic cell-based anticancer immunotherapy: maturation state does have clinical impact. *Cancer Immunology, Immunotherapy*.

[114] de Vries IJM, Lesterhuis WJ, Scharenborg NM (2003). Maturation of dendritic cells is a prerequisite for inducing immune responses in advanced melanoma patients. *Clinical Cancer Research*.

[115] Dhodapkar MV, Steinman RM, Krasovsky J, Munz C, Bhardwaj N (2001). Antigen-specific inhibition of effector T cell function in humans after injection of immature dendritic cells. *Journal of Experimental Medicine*.

[116] Jonuleit H, Kühn U, Müller G (1997). Pro-inflammatory cytokines and prostaglandins induce maturation of potent immunostimulatory dendritic cells under fetal calf serum-free conditions. *European Journal of Immunology*.

[117] Morse MA, Zhou LJ, Tedder TF, Kim Lyerly H, Smith C (1997). Generation of dendritic cells in vitro from peripheral blood mononuclear cells with granulocyte-macrophage-colony-stimulating factor, interleukin-4, and tumor necrosis factor-*α* for use in cancer immunotherapy. *Annals of Surgery*.

[118] Luft T, Jefford M, Luetjens P (2002). Functionally distinct dendritic cell (DC) populations induced by physiologic stimuli: prostaglandin E2 regulates the migratory capacity of specific DC subsets. *Blood*.

[119] Snijders A, Kalinski P, Hilkens CMU, Kapsenberg ML (1998). High-level IL-12 production by human dendritic cells requires two signals. *International Immunology*.

[120] Paustian C, Caspell R, Johnson T (2011). Effect of multiple activation stimuli on the generation of Th1-polarizing dendritic cells. *Human Immunology*.

[121] Mailliard RB, Wankowicz-Kalinska A, Cai Q (2004). *α*-type-1 polarized dendritic cells: a novel immunization tool with optimized CTL-inducing activity. *Cancer Research*.

[122] Dohnal AM, Witt V, Hügel H, Holter W, Gadner H, Felzmann T (2007). Phase I study of tumor Ag-loaded IL-12 secreting semi-mature DC for the treatment of pediatric cancer. *Cytotherapy*.

[123] Lehner M, Stilper A, Morhart P, Holter W (2008). Plasticity of dendritic cell function in response to prostaglandin E 2 (PGE2) and interferon-*γ* (IFN-*γ*). *Journal of Leukocyte Biology*.

[124] Boullart ACI, Aarntzen EHJG, Verdijk P (2008). Maturation of monocyte-derived dendritic cells with Toll-like receptor 3 and 7/8 ligands combined with prostaglandin E2 results in high interleukin-12 production and cell migration. *Cancer Immunology, Immunotherapy*.

[125] Sanchez PJ, McWilliams JA, Haluszczak C, Yagita H, Kedl RM (2007). Combined TLR/CD40 stimulation mediates potent cellular immunity by regulating dendritic cell expression of CD70 in vivo. *Journal of Immunology*.

[126] Iwamoto S, Ishida M, Takahashi K, Takeda K, Miyazaki A (2005). Lipopolysaccharide stimulation converts vigorously washed dendritic cells (DCs) to nonexhausted DCs expressing CD70 and evoking long-lasting type 1 T cell responses. *Journal of Leukocyte Biology*.

[127] Bontkes HJ, Kramer D, Ruizendaal JJ (2007). Dendritic cells transfected with interleukin-12 and tumor-associated antigen messenger RNA induce high avidity cytotoxic T cells. *Gene Therapy*.

[128] Bontkes HJ, Kramer D, Ruizendaal JJ, Meijer CJLM, Hooijberg E (2008). Tumor associated antigen and interleukin-12 mRNA transfected dendritic cells enhance effector function of natural killer cells and antigen specific T-cells. *Clinical Immunology*.

[129] Liu Y, Zhang X, Zhang W (2002). Adenovirus-mediated CD40 ligand gene-engineered dendritic cells elicit enhanced CD8^+^ cytotoxic T-cell activation and antitumor immunity. *Cancer Gene Therapy*.

[130] Hanks BA, Jiang J, Singh RAK (2005). Re-engineered CD40 receptor enables potent pharmacological activation of dendritic-cell cancer vaccines in vivo. *Nature Medicine*.

[131] Chen YX, Man K, Guang SL (2007). A crucial role for dendritic cell (DC) IL-10 in inhibiting successful DC-based immunotherapy: superior antitumor immunity against hepatocellular carcinoma evoked by DC devoid of IL-101. *Journal of Immunology*.

[132] Liu G, Ng H, Akasaki Y (2004). Small interference RNA modulation of IL-10 in human monocyte-derived dendritic cells enhances the Th1 response. *European Journal of Immunology*.

[133] Wobser M, Voigt H, Houben R (2007). Dendritic cell based antitumor vaccination: impact of functional indoleamine 2,3-dioxygenase expression. *Cancer Immunology, Immunotherapy*.

[134] Shen L, Evel-Kabler K, Strube R, Chen SY (2004). Silencing of SOCS1 enhances antigen presentation by dendritic cells and antigen-specific anti-tumor immunity. *Nature Biotechnology*.

[135] Evel-Kabler K, Song XT, Aldrich M, Huang XF, Chen SY (2006). SOCS1 restricts dendritic cells’ ability to break self tolerance and induce antitumor immunity by regulating IL-12 production and signaling. *Journal of Clinical Investigation*.

[136] Kao JY, Gong Y, Chen CM, Zheng QD, Chen JJ (2003). Tumor-derived TGF-*β* reduces the efficacy of dendritic cell/tumor fusion vaccine. *Journal of Immunology*.

[137] Caldwell CC, Kojima H, Lukashev D (2001). Differential effects of physiologically relevant hypoxic conditions on T lymphocyte development and effector functions. *Journal of Immunology*.

[138] Futalan D, Huang CT, Schmidt-Wolf IG (2011). Effect of oxygen levels on the physiology of dendritic cells: implications for adoptive cell therapy. *Molecular Medicine*.

[139] Vaupel P, Kallinowski F, Okunieff P (1989). Blood flow, oxygen and nutrient supply, and metabolic microenvironment of human tumors: a review. *Cancer Research*.

[140] Yang M, Ma C, Liu S (2009). Hypoxia skews dendritic cells to a T helper type 2-stimulating phenotype and promotes tumour cell migration by dendritic cell-derived osteopontin. *Immunology*.

[141] Wang Q, Liu C, Zhu F (2010). Reoxygenation of hypoxia-differentiated dentritic cells induces Th1 and Th17 cell differentiation. *Molecular Immunology*.

[142] Napoletano C, Pinto D, Bellati F (2007). A comparative analysis of serum and serum-free media for generation of clinical grade DCs. *Journal of Immunotherapy*.

[143] Lehner M, Morhart P, Stilper A, Holter W (2005). Functional characterization of monocyte-derived dendritic cells generated under serumfree culture conditions. *Immunology Letters*.

[144] de Vries IJM, Eggert AAO, Scharenborg NM (2002). Phenotypical and functional characterization of clinical grade dendritic cells. *Journal of Immunotherapy*.

[145] Duperrier K, Eljaafari A, Dezutter-Dambuyant C (2000). Distinct subsets of dendritic cells resembling dermal DCs can be generated in vitro from monocytes, in the presence of different serum supplements. *Journal of Immunological Methods*.

[146] Peng JC, Thomas R, Nielsen LK (2005). Generation and maturation of dendritic cells for clinical application under serum-free conditions. *Journal of Immunotherapy*.

[147] Lechmann M, Berchtold S, Steinkasserer A, Hauber J (2002). CD83 on dendritic cells: more than just a marker for maturation. *Trends in Immunology*.

[148] Engell-Noerregaard L, Hansen TH, Andersen MH, Thor Straten P, Svane IM (2009). Review of clinical studies on dendritic cell-based vaccination of patients with malignant melanoma: assessment of correlation between clinical response and vaccine parameters. *Cancer Immunology, Immunotherapy*.

[149] Murthy V, Moiyadi A, Sawant R, Sarin R (2009). Clinical considerations in developing dendritic cell vaccine based immunotherapy protocols in cancer. *Current Molecular Medicine*.

[150] Copier J, Bodman-Smith M, Dalgleish A (2011). Current status and future applications of cellular therapies for cancer. *Immunotherapy*.

[151] Copier J, Dalgleish AG, Britten CM (2009). Improving the efficacy of cancer immunotherapy. *European Journal of Cancer*.

[152] García F, Climent N, Assoumou L (2011). A therapeutic dendritic cell-based vaccine for HIV-1 infection. *Journal of Infectious Diseases*.

[153] García F, Lejeune M, Climent N (2005). Therapeutic immunization with dendritic cells loaded with heat-inactivated autologous HIV-1 in patients with chronic HIV-1 infection. *Journal of Infectious Diseases*.

[154] Lu W, Arraes LC, Ferreira WT, Andrieu JM (2004). Therapeutic dendritic-cell vaccine for chronic HIV-1 infection. *Nature Medicine*.

[155] Giermasz AS, Urban JA, Nakamura Y (2009). Type-1 polarized dendritic cells primed for high IL-12 production show enhanced activity as cancer vaccines. *Cancer Immunology, Immunotherapy*.

[156] Lee JJ, Foon KA, Mailliard RB, Muthuswamy R, Kalinski P (2008). Type 1-polarized dendritic cells loaded with autologous tumor are a potent immunogen against chronic lymphocytic leukemia. *Journal of Leukocyte Biology*.

[157] Kalinski P, Urban J, Narang R, Berk E, Wieckowski E, Muthuswamy R (2009). Dendritic cell-based therapeutic cancer vaccines: what we have and what we need. *Future Oncology*.

[158] Czerniecki BJ, Koski GK, Koldovsky U (2007). Targeting HER-2/neu in early breast cancer development using dendritic cells with staged interleukin-12 burst secretion. *Cancer Research*.

[159] Traxlmayr MW, Wesch D, Dohnal AM (2010). Immune suppression by *γδ* t-cells as a potential regulatory mechanism after cancer vaccination with IL-12 secreting dendritic cells. *Journal of Immunotherapy*.

[160] van Nuffel AM, Benteyn D, Wilgenhof S (2012). Intravenous and intradermal TriMix-dendritic cell therapy results in a broad T-cell response and durable tumor response in a chemorefractory stage IV-M1c melanoma patient. *Cancer Immunology, Immunotherapy*.

[161] Wilgenhof S, van Nuffel AMT, Corthals J (2011). Therapeutic vaccination with an autologous mRNA electroporated dendritic cell vaccine in patients with advanced melanoma. *Journal of Immunotherapy*.

[162] Bonehill A, van Nuffel AMT, Corthals J (2009). Single-step antigen loading and activation of dendritic cells by mRNA electroporation for the purpose of therapeutic vaccination in melanoma patients. *Clinical Cancer Research*.

[163] Liu Q, Zhang C, Sun A, Zheng Y, Wang L, Cao X (2009). Tumor-educated CD11bhighIalow regulatory dendritic cells suppress T cell response through arginase I. *Journal of Immunology*.

[164] Gottfried E, Kreutz M, Mackensen A (2008). Tumor-induced modulation of dendritic cell function. *Cytokine and Growth Factor Reviews*.

[165] Buelens C, Willems F, Delvaux A (1995). Interleukin-10 differentially regulates B7-1 (CD80) and B7-2 (CD86) expression on human peripheral blood dendritic cells. *European Journal of Immunology*.

[166] Enk AH, Angeloni VL, Udey MC, Katz SI (1993). Inhibition of Langerhans cell antigen-presenting function by IL-10: a role for IL-10 in induction of tolerance. *Journal of Immunology*.

[167] Cunningham AL, Donaghy H, Harman AN, Kim M, Turville SG (2010). Manipulation of dendritic cell function by viruses. *Current Opinion in Microbiology*.

[168] Liu B, Woltman AM, Janssen HLA, Boonstra A (2009). Modulation of dendritic cell function by persistent viruses. *Journal of Leukocyte Biology*.

[169] Beck K, Meyer-König U, Weidmann M, Nern C, Hufert FT (2003). Human cytomegalovirus impairs dendritic cell function: a novel mechanism of human cytomegalovirus immune escape. *European Journal of Immunology*.

[170] le Bon A, Etchart N, Rossmann C (2003). Cross-priming of CD8^+^ T cells stimulated by virus-induced type I interferon. *Nature Immunology*.

[171] Dubsky P, Saito H, Leogier M (2007). IL-15-induced human DC efficiently prime melanomaspecific naive CD8^+^ T cells to differentiate into CTL. *European Journal of Immunology*.

[172] Klechevsky E, Morita R, Liu M (2008). Functional specializations of human epidermal langerhans cells and CD14^+^ dermal dendritic cells. *Immunity*.

[173] Letvin NL (2007). Correlates of immune protection and the development of a human immunodeficiency virus vaccine. *Immunity*.

[174] Palucka K, Ueno H, Zurawski G, Fay J, Banchereau J (2010). Building on dendritic cell subsets to improve cancer vaccines. *Current Opinion in Immunology*.

[175] O’Keeffe M, Hochrein H, Vremec D (2002). Effects of administration of progenipoietin 1, Flt-3 ligand, granulocyte colony-stimulating factor, and pegylated granulocyte-macrophage colony-stimulating factor on dendritic cell subsets in mice. *Blood*.

[176] Naik SH, Proietto AI, Wilson NS (2005). Cutting edge: generation of splenic CD8^+^ and CD8^−^ dendritic cell equivalents in Fms-like tyrosine kinase 3 ligand bone marrow cultures. *Journal of Immunology*.

[177] Brasel K, de Smedt T, Smith JL, Maliszewski CR (2000). Generation of murine dendritic cells from flt3-ligand-supplemented bone marrow cultures. *Blood*.

